# Annual Address of the President before the Medical Society of the County of Erie

**Published:** 1894-03

**Authors:** John Parmenter

**Affiliations:** 116 North Pearl Street; Buffalo, N. Y.


### Buffalo Medical and Surgical Journal

Vol. XXXIII. MARCH, 1894. No. 8.

### Original Address.

### ANNUAL ADDRESS OF THE PRESIDENT BEFORE THE MEDICAL SOCIETY OF THE COUNTY OF ERIE.

*January 9, 1894.*

By JOHN PARMENTER, M. D., Buffalo, N. Y.

Before I begin the few words that I have to say to you on retiring from the presidency of this Society, I wish to thank you most cordially for the honor conferred upon me by making me your president. It was my misfortune to be absent from the meeting one year ago, when I was so generously and handsomely remembered by you, and, hence, I could not give you then the thanks and words of appreciation that I now take occasion to do. And yet, while I am deeply appreciative of the honor of being the presiding officer of this ancient body, I cannot refrain from expressing the belief that the best interests, the dignity and the usefulness of this organization would be better maintained and promoted by the election to the presidency of some one of its many members who have "grown gray in service," with all that that term means in judgment and executive ability. It seems to me that such a policy for the future would be a safe and wise one. Many of our best known and respected brother practitioners from adjoining towns should have recognition in this way. It would be a graceful act for the voting majority, composed of city member,s to remember this in the nomination and election of future officers of this society. And right here let me call the attention of the society to the fact that during the past year an unusual amount of work connected with the revision of the by-laws has been performed by the secretary, and that he should receive a liberal compensation for the same. Both the secretary and the treasurer are officers who give the society an amount of time and labor that merits a compensation which they never receive. I sincerely hope that the society will see this matter in the just and true light, and act accordingly.

The events of our society meetings during the past two or three years have suggested to my mind very forcibly the differences between our society in this period and the same a decade ago, when I first became a member of it. In the first years of my membership, the chief functions were political in character, and the politics of that order which dealt not with the larger and more important issues of the day, but rather that petty kind which made many entertain unpleasant feelings toward their brothers and distracted all from the legitimate and proper work of the society. If the record of the past two or three years is an index of the future, that day is past. Henceforth, personalities, lobbying for office, impeachment of hospital reports and the like will give place to the reading and discussion of scientific papers, the endorsement of laudable public measures, and the promotion of higher ethical standards among its members. We have already, as I have said, in the past two or three years begun the good work. We have listened to the reading of papers by men eminent in their chosen lines, and these have been discussed in a manner creditable to the membership of any society; we have endorsed the important measures which have come before us for our recognizance and aid; we have given our hearty support to the efforts of the state in making a higher standard of medical education; we have maintained a successful opposition to the quackery which has so long infested our city, and we have done one other thing which, to my mind, marks an era in the history of this society, viz.: we have joined hands with our homeopathic colleagues and made a united effort in a common cause to effect a desired result. This means a great deal; it means the first battering-ram against the high and solid wall which has so long separated us from our homeopathic brethren; it means the beginning of the end of schools; it means the inauguration of a time when we shall all be physicians, free from any dogma, to treat the sick according to the best dictates of reason and science. The mere existence of a dividing line leads one to inquire why it is there, and the question as the matter stands today is one not easy to answer. There are so many points of similarity in the theory and practice of physic that are believed and practised by both alike, that one is made to wonder that men engaged in such serious and responsible scientific work will longer permit themselves to be divided by any such hair-splitting processes as have held sway in the past. We need but to look over the curricula of the various homeopathic medical colleges in this country to find therein a place of instruction which differs but little from that of our own schools. In almost all branches, even in therapeutics, the same text-books find a place in the list of books recommended to students. Thus, in the announcement of the Homeopathic Hospital College of Cleveland, O., the following are some of them:

BOOKS RECOMMENDED FOR STUDY.

| | TEXT-BOOKS. | REFERENCES. |
|---|---|---|
| SURGERY | *Helmuth* | Wyeth, Gross, Stephen Smith; Gerster, Agnew. |
| ANATOMY | *Descriptive—Morris. Osteology—Holden. Surgical Anatomy—Sheild.* | Gray, Holden's Manual, McClellan's Regional. Treves. |
| PRACTICE OF MEDICINE, GENERAL AND SPECIAL PATHOLOGY AND DIAGNOSIS | *Raue's Pathology and Diagnosis; Baehr's Therapeutics; Ardent's System of Medicine; Ockford's Handbook of Practice* | Clapp's Tabular Handbook of Auscultation and Percussion; Gray's Diseases of Nervous System; Da Costa on Diagnosis; Loomis' Theory and Practice of Medicine; Pepper's American Text-book of Theory and Practice. |
| MATERIA MEDICA | *Hering; Dunham's Lectures; Hughe's; Farrington; The Organon* | Hahnemann; Hempel; Salient Materia Medica, Cleveland; Allen's Cyclopedia. |
| PHYSIOLOGY | *Kirke* | Yeo; Landois; Foster. |
| OBSTETRICS | *Guernsey; Leavitt* | Playfair; Lusk; Fordyce Barker on Puerperal Diseases; Keating. |
| GYNECOLOGY | *Ludlam* | Hart and Barbour's Manual of Gynecology; Pozzi. |
| DISEASES OF CHILDREN | *Guernsey; Teste* | Keating. |
| CHEMISTRY AND TOXICOLOGY | *Simon; Pellew; Leffmann and Beam; Practical Chemistry* | Fown's Chemistry; Taylor on Poisons; Beale on Urinary Deposits. |
| OPHTHALMOLOGY | *Angell; Buffum; Morton; Noyes or Schweinitz.* | |

| | TEXT-BOOKS. | REFERENCES. |
|---|---|---|
| OTOLOGY | *Winslow* | Houghton or St. John Roosa. |
| DERMATOLOGY | *Kippax* | Saunder's Compend.; Byford. |
| MICROSCOPY | *Delafield's Elements of Histology; Klein* | Practical Microscopy, Miller; The Microscope in Medicine, Beale. |
| MEDICAL JURISPRUDENCE | *The Code of Medical Ethics; Vol. I. Greenleaf on Evidence of Malpractice, Medical Evidence and Insanity, by Ewell; Vol. III. Legal Medicine by Chas. M. Tidy.* | |
| SANITARY SCIENCE | *Parks* | Buck, Wilson. |
| DISEASES OF NOSE AND THROAT | *Sajous: Bosworth* | Lennox Brown, 3d Ed. |
| ORIFICIAL SURGERY | *Pratt.* | |
| LEXICON | *Gould; Thomas.* | |

We see, therefore, that the same training is given the students who are to become practitioners of medicine under the one or the other banner.

Again, when we consider the selection and administration of the drug to be given, we shall find a marked similarity. Members of the homeopathic and the regular professions choose their drugs in accordance with their best judgment as to its physiological effects and its therapeutic applications in disregard of whether it belongs to the category of drugs known as homeopathic or not. In short, the members of both professions enjoy and exercise full liberty in planning and executing their treatment of their patients. That all this is true is easily shown by reference to the statements of medical authorities in the homeopathic profession, either in their text-books, in their editorial remarks, or in their sayings in society meetings. Thus, a resolution of the Homeopathic Medical Society of New York, passed February 8, 1878, declares that

A belief in the laws of similars does not debar us (homeopathic physicians) from recognizing and making use of the results of any experience, and we shall exercise and defend the inviolable right of every educated physician to make practical use of any established principle of medicine, or of any therapeutic facts founded on experiments and verified by experience, so far as in his individual judgment they shall tend to promote the welfare of those under his professional care.

The New York *Medical Times* (May, 1892, page 48,) says in an editorial as follows:

It is apparent to even the casual observer, that scientific study is rapidly bringing all schools more in harmony with each other, and while it eliminates more and more the theoretical and conjectural, is building up a scientific therapeutics based upon the unanswerable logic of facts, the general outline of which will be acceptable to all. The same journal further suggests that if the societies composed of non-sectarian physicians revise their by-laws so that physicians now called homeopaths may be eligible for membership, the homeopathic medical societies should then drop their sectarian name.

This is a most important suggestion, and I shall ask you to bear it in mind again in a few moments.

Another recognized journal of homeopathy (*The Homeopathic News*, March, 1892), in a stirring editorial, says as follows:

We venture to assert that had not our school drifted away from the practice of forty years ago, it would have been dead and buried long since.

Continuing, this recognized journal of homeopathy says:

We have drifted away from the practice of giving a pellet of the two-hundredth or higher, and waiting thirty or sixty days for its curative effects ; from the prescribing of a high dilution by smelling the dry pellets, those same pellets "grafted" by shaking a thousand pure pellets with one medicated by the ten-thousandth.

We have drifted away from a belief in provings made by taking a single dose of the one-thousandth, thirtieth, or third even, and then recording all the symptoms felt by the prover—natural symptoms, colds, diarrhea, etc., for the next sixty days!

We have drifted away from the carrying a pocket repertory to the bedside of the patient, and recording the symptoms in columns, and a weary search in said repertory until a mechanical similimum was found.

We have drifted away from the days when our pseudo-surgery was a disgraceful farce, when we expected silica to open a felon, or hepar sulphur to lance an abscess.

We have drifted away from the narration of miraculous cures with the highest attenuations, which were not cures at all, but a spontaneous finale of a self-limited disease.

We have drifted from the days when our practitioners would sit by the bedside of a woman dying of uterine hemorrhage, hunting in a repertory for the "indicated remedy," while the vital fluid was ebbing away, without recourse to the tampon or ergot.

I have made these quotations to show you that homeopathy as taught by Hahnemann and as practised today, are quite different things, and that the intelligent homeopathic practitioners of the present time are fast receding from the position maintained by their predecessors. They too, like us, give hygiene and diet a most important place in their therapeutics. They, too, use palliative measures in painful and incurable diseases. Thus we see how much alike are the opinions and practices of two sections of the medical professions which today are separated by a dividing line that seems to have no justification for its existence.

In Buffalo I am glad to say that already the entering wedge has been employed, and we now have consummated the first step toward a union. I refer to the recent organization of a staff for the Erie County Hospital, upon which serve physicians and surgeons from the regular and the homeopathic ranks. Such a combination has never before occurred in any institution in this city, so far as I know; and although sufficient time has not yet elapsed to judge of the efficiency and harmony of this joint staff, I have no doubt from the liberal views and general broad-minded character of our homeopathic confrères that the result will be in every way a complete justification of the wisdom and liberality of our Board of Supervisors, who have brought all this to pass. Now, can we not go a step farther—a long and important step? Can we, the Erie County Medical Society, not declare ourselves to be free from the bigotry and narrow-mindedness of the past and show ourselves willing to subjugate all former prejudices by earnestly urging for a union of the regular and homeopathic physicians of the County of Erie? In short, to do all we can to bring about the amalgamation of the Erie County Homeopathic Medical Society with our own, three pertinent questions arise:

1. Is such union feasible?
2. If so, is it desirable?
3. How can it be effected?

Regarding the first question, from what has already been said, it will, I think, be apparent that but few differences between us really exist, and these by no means vital, and this means harmony. I have already quoted the resolutions of the New York State Homeopathic Medical Society, and I repeat them right here, that they may be firmly fixed in your minds:

> . . . . The belief in the law of similars does not debar us (homeopathic physicians) from recognizing and making use of the results of any experience, and we shall exercise and defend the inviolable right of every educated physician to make practical use of any established principle of medical science, or of any therapeutic facts founded on experiments and verified by experience, so far as in his individual judgment they shall tend to promote the welfare of those under his professional care,

What difference, I ask, is there, then, between the members of the above mentioned society and ourselves? Any homeopathic physician subscribing to the resolutions just quoted is eligible for membership in any medical society composed of members like ourselves. This means, to my mind, that so far as common ideas and practices can make it, the amalgamation is feasible.

Is it desirable? In every other function in our lives we meet and work together. In the church, in politics, in philanthropy, we unite our efforts in friendly spirit to bring about necessary or desired ends; we meet as equals in the drawing-room and all of us number some of them among our best and most respected friends. When in one or the other of the above-mentioned causes we band together, we hasten and render more certain the accomplishment of these causes, because in union there is strength. So it would be in our life-work for medical science. Here, too, union would be strength and our united efforts would the sooner make us as a profession better in every way. The regular school by no means knows it all. Homeopathy has taught us many valuable lessons. It has taught us the curative power of unaided Nature, the use of diet and regimen in treating disease, and the uselessness, even harm, of giving powerful drugs in many instances, and the undesirability of "shotgun" prescriptions which combine many and nasty medicines upsetting alike to stomach and health. And we have learned these lessons, too, not as friendly counsel from co-workers in science, but because it was true and we were forced to adopt the practices advised as a child takes bitter medicine, not for its sweet taste, but because it does good. If this be so, how much time and knowledge might we not have gained had these acquisitions to our knowledge come from friends instead of competitors. Again, is not the man himself a better man? Is he not more tolerant and liberal-minded to his brethren? Is he not a better physician to his patient when willing to accept from any authoritative source any useful additions to his professional knowledge? My answer then is, it is desirable because by union we make our profession stronger and better, we make ourselves as men more liberal and tolerant with all that these adjectives mean, and, finally, we subserve the best interests of our patients.

Do not misunderstand me as defending homeopathy as such. I have nothing but contempt for doctrines which teach that the rule *similia similibus curantur* should be elevated to the dignity of a regular law, that there is a curative power in infinitesimal doses, and that this power increases as the dose of the drug decreases. What I do say is that, incidentally, homeopathy, so-called, has done good as above stated and that we have adopted some of the lessons and that it is not unlikely that there are some more for us to learn.

Now to our third question: How can we bring this about? Inasmuch as we are the older and the numerically stronger body, it would, it seems to me, be a graceful and proper act for this society to ask the Erie County Homeopathic Medical Society to join with us in making one large, strong body of physicians, who should be co-workers in medicine, disclaiming all 'pathy, united in action for the betterment of their science and for the alleviation of mankind. To attain this end, one thing, at least, is essential. Our neighbors must discard the name homeopathic. From what I have read, some quotations you have already heard, and from conversation with members of the homeopathic profession, here and elsewhere, I am convinced that but few true disciples of Hahnemann remain. Anyone who regards the rule *similia similibus curantur* as of partial and not universal application is no longer a homeopath and has no right to call himself as such. I hazard the conjecture that the very great majority of homeopaths, so-called, in this city are not such, and that to put aside the name would not necessarily tug hard at their heart-strings. At any rate, if we make the offer under the limitations stated, we have shown our desire to throw away the narrow and belittling traditions of the past and to do our utmost to promote universal brotherhood. This is an opportune time. A new year and a new era together would be added to the history of this venerable and important society.

116 North Pearl Street.

---

Potassium Permanganate an Antidote for Morphine.—Recent despatches state that Dr. William Moore, of New York, claims that when morphine is taken into the stomach and promptly followed by the ingestion of a somewhat larger dose of potassium permanganate, the latter drug causes the oxidation of the alkaloid, rendering it harmless. To demonstrate the correctness of his view, he recently, in the presence of a number of physicians, swallowed three grains of morphine, taking immediately afterwards, four grains of potassium permanganate; and experienced no bad effects.—*Philadelphia Polyclinic*, February 7, 1894.

